# Telerehabilitation for Family Caregivers of Stroke Survivors: A Systematic Review and Meta-Analysis

**DOI:** 10.1155/2023/3450312

**Published:** 2023-06-14

**Authors:** Wen-Jing Sun, Yuan-Yuan Song, Cong Wang, Yan Jiang, Wen-Yao Cui, Wen-Jie Liu, Yan Liu

**Affiliations:** ^1^Department of Neurosurgery, West China Hospital, Sichuan University/West China School of Nursing, Sichuan University, Chengdu, China; ^2^Evidence-Based Nursing Center, West China Hospital, Sichuan University/West China School of Nursing, Sichuan University, Chengdu, China; ^3^Intensive Care Unit, West China Hospital, Sichuan University/West China School of Nursing, Sichuan University, No. 37 Guoxue Alley, Wuhou District, Chengdu, Chengdu, Sichuan 610041, China; ^4^Department of Nursing, West China Hospital, Sichuan University/West China School of Nursing, Sichuan University, Chengdu, China

## Abstract

**Aim:**

This systematic review aimed at evaluating the effectiveness of telerehabilitation on family caregivers of stroke survivors.

**Background:**

After discharge from the hospital, family caregivers of stroke survivors faced physical and psychological stress. Telerehabilitation seems crucial for family caregivers. However, the impact of telerehabilitation on family caregivers' health outcomes remains to be studied. *Evaluation*. Six databases (PubMed, Embase, Cochrane Library, Web of Science, CINAHL, and PsycINFO) were searched up to June 16^th^, 2022, without language restrictions. The Revised Cochrane Risk-of-bias Tool for Randomized Trials was used to assess the quality of included studies. The GRADEpro (Grading of Recommendations Assessment, Development, and Evaluation Profile) tools were applied to assess the synthesized evidence quality. The subgroup analysis was performed according to the intervention formats. Statistical analysis was conducted using Review Manager 5.3, and the publication bias was calculated by Stata 14.0. *Key Issue(s)*. A total of 16 studies containing 992 caregivers were pooled in this systematic review. Telerehabilitation significantly improved the caregiver burden (SMD = −0.18, 95% CI = −0.35∼−0.02, *P*=0.03, moderate-quality evidence), knowledge (SMD = 0.75, 95% CI = 0.03∼1.47, *P*=0.04, very low-quality evidence), and competence (SMD = 1.35, 95% CI = 0.82∼1.88, *P* < 0.001, very low-quality evidence) but not depression (SMD = −0.04, 95% CI = −0.3∼0.21, *P*=0.74, moderate-quality evidence), anxiety (MD = 0.68, 95% CI = −0.68∼2.04, *P*=0.32, low-quality evidence), and self-efficacy (SMD = −0.30, 95% CI = −1.22∼0.61, *P*=0.52, very low-quality evidence) in family caregivers of stroke survivors. The subgroup analysis demonstrated that multi-form telerehabilitation (SMD = 1.86, 95% CI = 1.32∼2.40, *P* < 0.001) was significantly effective in improving caregiving competence.

**Conclusion:**

Telerehabilitation can effectively reduce the caregiver burden as well as improve the knowledge and competence of stroke caregivers. *Implications for Nursing Management*. The emergence of telerehabilitation can help relieve caregivers' stress and provide a new form for nursing managers to make discharge plans for stroke.

## 1. Introduction

Stroke remains the second-leading cause of death and the third-leading cause of death and disability combined worldwide [1]. It is estimated that the total global direct and indirect costs of stroke are $891 ($746∼1077) billion, accounting for 1.12% of the global gross domestic product [2]. Almost 70%–80% of stroke survivors have complications such as cognitive impairment, motor dysfunction, and swallowing dysfunction [3]. In addition, 25%–50% of stroke survivors become partially or entirely dependent on others in activities of daily living [4]. According to a recent systematic review, the average medical cost of a stroke family is about 5,798.15 to 140,048 euros [5]. A series of stroke complications, massive demand for rehabilitation, and heavy economic pressure may overwhelm stroke survivors and family caregivers.

After discharge from the hospital, family caregivers such as parents, spouses, children, and siblings provide informal and unpaid care for stroke survivors. The lack of professional guidance and reasonable rehabilitation plans often lead to poor knowledge, belief, and behavior for caregivers and poor home rehabilitation effects for stroke survivors [6]. According to a survey, 82% of family caregivers provided stroke care for more than 8 hours a day [7]. This challenging care may bring adverse outcomes to family caregivers' physical and mental health. A longitudinal study in Singapore showed that the health status of stroke caregivers gradually declined within 12 months after patients suffered a stroke [8]. Approximately 21.4% (95% [Confidence Interval, CI]: 11.6%∼35.9%) of family caregivers experienced anxiety symptoms, while 40.2% (95% CI: 30.1%∼51.1%) experienced depression symptoms during the period of caring for stroke survivors [9]. The vast majority of family caregivers felt lifestyle changes and faced varying degrees of caregiving burden [7]. To solve these problems, routine rehabilitation is carried out for stroke caregivers including on-site and face-to-face guidance such as outpatient follow-up, home visits, etc. [10]. Routine rehabilitation could help doctors comprehensively evaluate patients through visual touch and listening. However, according to a survey, stroke caregivers need to spend $1,167 on healthcare services, resulting in an increase of $726 in total home care costs. [11]. In addition, routine rehabilitation makes it difficult for nurses to effectively supervise the rehabilitation effects, resulting in discontinuity of care and adverse effects on stroke survivors and caregivers.

Therefore, the implementation of remote interventions may help satisfy the needs of family caregivers, providing them with stroke-related knowledge and skills to adapt to life and role changes. Telerehabilitation uses information and communication technology (telephone, mail, and video conferencing) and computer science technology (applets, websites, and mobile applications) to provide services remotely [12]. According to a practice guideline published by American Congress of Rehabilitation Medicine Stroke International Special Interest Group, telerehabilitation could benefit family caregivers in several ways, including (1) reduction of direct and indirect costs: despite the high costs of placing network devices at home, telerehabilitation reduces outpatient and round-trip costs; (2) breaking geographical restrictions: it provides more opportunities for patients in rural and remote areas and improves the accessibility of medical services; (3) providing prompt diagnosis and quick response: experts can use communication technology to achieve timely consultation and treatment; (4) reduction in the spread of infectious diseases such as COVID-19 [13].

Despite the various advantages of telerehabilitation, current research focuses too much on patient health outcomes, resulting in caregivers often being neglected. Multiple systematic reviews have demonstrated the effectiveness of telerehabilitation in enhancing patients' motor function, generic quality of life, and activities of daily living [14, 15]. However, the vulnerability and sensitivity of stroke caregivers still deserve attention. There have been randomized controlled trials (RCTs) comparing the differences between telerehabilitation and routine rehabilitation for stroke caregivers [16–18]. Conflicting evidence on caregiver health outcomes has led to a lack of consistent findings and difficulties for healthcare practitioners to make appropriate judgments [19, 20]. Therefore, this study evaluated and synthesized evidence to explore the effectiveness of telerehabilitation on the health outcomes of stroke caregivers.

## 2. Materials and Methods

This systematic review was conducted according to the Preferred Reporting Items for Systematic Reviews and Meta-Analyses (PRISMA) 2020 checklist and was registered in PROSPERO (https://www.crd.york.ac.uk/prospero/) with a registration number of CRD42022358364.

### 2.1. Search Strategy

Six databases were comprehensively searched, including PubMed, Embase, Cochrane Library, Web of Science, CINAHL, and PsycINFO. Databases were searched up to June 16^th^, 2022, without any language restrictions. The search strategy was designed based on the PICOS framework (e.g., P: stroke, family caregiver, informal caregiver, and carer). Free terms were employed to supplement the MeSH terms. The references of the included studies were manually searched to identify any additional studies. The complete list of search strategies is listed in Supplementary Table 1.

### 2.2. Eligibility Criteria

The inclusion criteria were as follows: (1) stroke survivors aged ≥18 years; (2) family caregivers such as parents, spouses, children, and siblings provided home care for stroke survivors; (3) telerehabilitation was applied to family caregivers of stroke survivors to provide them with caregiving skills or improve their health outcomes; (4) the interventions may include videoconferencing, telephone sessions, applets, virtual reality, etc., and the routine rehabilitation in the control group may include on-site and face-to-face rehabilitation; (5) outcomes reported caregiver burden, competence, knowledge, depression, anxiety, or self-efficacy; (6) RCTs. The exclusion criteria were as follows: (1) conference abstracts and posters; (2) protocols did not report results; (3) family caregivers received both telerehabilitation and on-site rehabilitation.

### 2.3. Study Selection and Data Extraction

Firstly, two reviewers used EndNote X9 to screen the searched records back-to-back and compared the records to reach an agreement. Any disagreements were resolved by a third reviewer. Secondly, two reviewers completed the data extraction independently. The extracted contents included author, year, country, sample size, age of patients, age of caregivers, interventions, intervention format, intervention duration, and outcomes. Lastly, the extracted results were cross-checked by two reviewers to determine the final results. Caregiver burden was identified as the primary outcome, while caregiving competence, knowledge, depression, anxiety, and self-efficacy were identified as secondary outcomes. The definition and evaluation methods of each outcome were elaborated in the previous protocol [21].

### 2.4. Quality Assessment

Two reviewers utilized the Revised Cochrane Risk-of-Bias Tool for Randomized Trials (ROB 2) to assess the quality of the included studies from five domains, including the randomization process, deviations from the intended interventions, missing outcome data, measurement of the outcome, and selection of the reported results. Overall bias was calculated according to the algorithm provided by the Cochrane Collaboration [22]. All disagreements were resolved by an expert in evidence-based medicine.

### 2.5. Data Synthesis and Analysis

Review Manager 5.3 was used to conduct the systematic review and meta-analysis. Since all outcomes in this study were continuous variables, the mean difference (MD) or standardized mean difference (SMD) with 95% CI was used to calculate the effects according to whether the same scale was employed. Data available from the included studies were processed to estimate missing values. The *P* value less than 0.05 was considered statistically significant. The *I*^2^ statistic was used to measure the heterogeneity of the included studies. The fixed effects model was utilized only if *I*^2^ ≤ 50% with *P* > 0.1; otherwise, the random effects model was utilized. The subgroup analysis was used to compare different intervention formats (single-form telerehabilitation and multiform telerehabilitation). The sensitivity analysis was conducted to test the robustness of results by changing effects model. The single-form telerehabilitation was defined as the use of only one remote intervention for stroke caregivers, while multiform telerehabilitation used two or more remote interventions for stroke caregivers, including network, videoconferencing, telephone sessions, applets, virtual reality, etc. The GRADEpro (Grading of Recommendations Assessment, Development, and Evaluation Profile) Guideline Development Tool (GDT) was applied to assess the quality of synthesized evidence. When at least ten studies reported the same outcome, the publication bias was evaluated using Stata 14.0. Egger's test with *P* < 0.1 considered the presence of publication bias.

## 3. Results

### 3.1. Search Results and Selection

**Figure 1:**
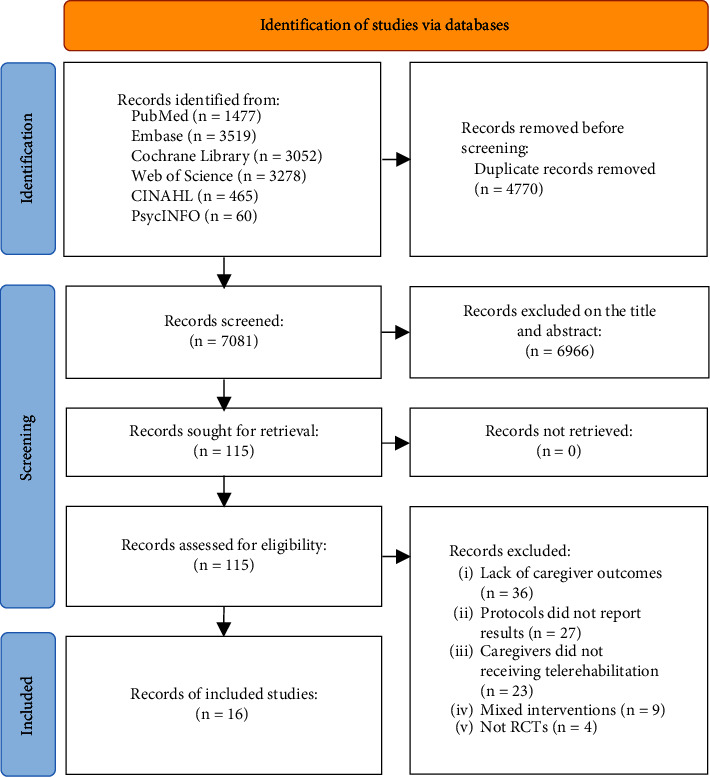
Flow diagram of study selection.

There were 11,851 identified via six databases. No additional records were identified by manual search. After duplicated records were removed, 7081 records were sought for retrieval. Later, 6966 unrelated records, 36 records without caregiver outcomes, 27 protocols without results, 23 records did not receive telerehabilitation, 9 records received mixed interventions, and 4 non-RCTs were excluded. According to the eligibility criteria, three three-arm studies [16, 18, 23] and three four-arm studies [24–26] removed redundant arms. A total of 16 RCTs were pooled in this systematic review finally. The flow diagram of study selection is shown in Figure 1.

### 3.2. Study Characteristics

The characteristics of the included studies are described in Table 1. A total of 16 RCTs containing 992 caregivers were pooled in this systematic review. The included studies were published between 2007 and 2021 in America (*n* = 5), China (*n* = 3), Korea (*n* = 2), Australia (*n* = 2), Netherlands (*n* = 2), New Zealand (*n* = 1), and Malaysia (*n* = 1). One article was published in Korean [16], one in Chinese [20], and the others in English. Although one study was a study protocol registered in the United States National Library of Medicine, it reported detailed results and was therefore included in this systematic review [24]. The intervention formats of telerehabilitation varied among all the studies. The main intervention formats included web-based software, websites, e-mail, DVD, telephone sessions, video conferencing, and applets. Six studies used multiple intervention formats of telerehabilitation [17, 19, 20, 26, 27, 32], while the rest used only one intervention format [10, 16, 18, 23–25, 28–31].

### 3.3. Quality Assessment

According to ROB 2, the quality assessment of each study is displayed in Supplementary Figure 1. Of the 16 RCTs, 2 studies (12.5%) were at a low risk, 4 studies (25%) were at a high risk, and the rest (62.5%) remained some concerns. The summary evidence of a bias risk for each outcome is detailed in Supplementary Table 2. The GRADEpro GDT determined the moderate quality of synthesized evidence on caregiver burden and depression symptoms. Synthesized evidence of anxiety was rated as low quality. The evidence of caregiving knowledge, competence, and self-efficacy was rated as very low quality. Studies with a risk of bias and a small sample size were the main factors affecting the evaluation results.

### 3.4. Meta-Analysis of the Outcomes

#### 3.4.1. Caregiver Burden

**Figure 2:**
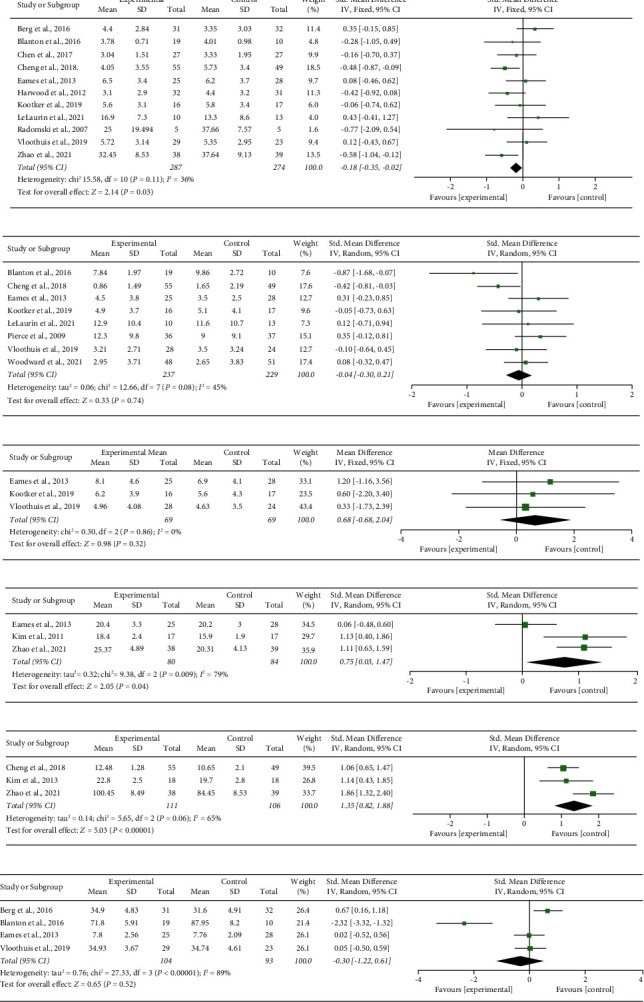
Forest plot of each outcome. (a) Caregiver burden. (b) Depression. (c) Anxiety. (d) Caregiving knowledge. (e) Caregiving competence. (f) Self-efficacy.

Twelve studies applied four scales (Caregiver Burden Index, Expanded Caregiver Burden Index, Zarit Burden Interview, and the Zarit Burden Interview-Short Form) to assess caregiver burden. Among these studies, one study treated caregiver stress as a classified variable, with 20.5% of caregivers in the intervention group and 34.8% of caregivers in the control group at a high-stress level [10]. Finally, 11 studies were enrolled in the meta-analysis. The fixed-effects model in Figure 2(a) shows that the caregiver burden is significantly lower in the telerehabilitation group than in the routine rehabilitation group (SMD = −0.18, 95% CI = −0.35∼−0.02, *P*=0.03, *I*^2^ = 36%). Sensitivity analysis showed that the results were not significant after changing into the random effects model (SMD = −0.16, 95% CI = −0.38∼0.06, *P*=0.15, *I*^2^ = 36%). The subgroup analysis in Supplementary Figure 2a shows that compared with routine rehabilitation, single-form telerehabilitation yields lower caregiver burden (SMD = −0.35, 95% CI = −0.58∼−0.12, *P*=0.003, *I*^2^ = 0%). There was no statistical difference between multiform telerehabilitation and routine rehabilitation in improving caregiver burden (SMD = 0.04, 95% CI = −0.34∼0.42, *P*=0.84,*I*^2^ = 58%). Egger's test observed no publication bias (*P*=0.453).

#### 3.4.2. Depression

Eight studies applied four scales (Center for Epidemiologic Studies Depression Scale, 10-item Center for Epidemiological Studies for Depression Scale, Hospital Anxiety and Depression Scale, and Patient Health Questionnaire-9) to assess depression symptoms. Because *I*^2^ < 50% with *P* < 0.1, the random-effects model was used for a conservative estimate of pooled data. The meta-analysis in Figure 2(b) shows that the telerehabilitation group has no significant effect on depression symptoms to the routine rehabilitation group (SMD = −0.04, 95% CI = −0.3∼0.21, *P*=0.74, *I*^2^ = 45%). Sensitivity analysis showed that the result was stable. The subgroup analysis in Supplementary Figure 2b shows that compared with routine rehabilitation, neither single-form rehabilitation (SMD = −0.26, 95% CI = −0.63∼0.12, *P*=0.18, *I*^2^ = 50%) nor multiform rehabilitation was significant in improving depression symptoms (SMD = 0.20, 95% CI = −0.08∼0.47, *P*=0.17, *I*^2^ = 0%).

#### 3.4.3. Anxiety

Three studies applied the Hospital Anxiety and Depression Scale to assess anxiety symptoms. Thus, MD was used to calculate the effect size. The fixed-effects model in Figure 2(c) shows that the telerehabilitation group has no significant effect on anxiety symptoms to the routine rehabilitation group (MD = 0.68, 95% CI = −0.68∼2.04, *P*=0.32, *I*^2^ = 0%).

#### 3.4.4. Caregiving Knowledge

Three studies applied different scales (Kong's Caregiving Knowledge Scale, Knowledge of Stroke Questionnaire, and Comprehensive Competence Assessment Questionnaire for Stroke Caregivers) to assess caregiving knowledge. The random-effects model in Figure 2(d) shows that the caregiving knowledge in the telerehabilitation group is significantly higher than in the routine rehabilitation group (SMD = 0.75, 95% CI = 0.03∼1.47, *P*=0.04, *I*^2^ = 79%). The high heterogeneity may result from different measurement tools.

#### 3.4.5. Caregiving Competence

Three studies applied different scales (Six-item Care Giving Mastery Scale, Caregiving Competence Scale, and Comprehensive Competence Assessment Questionnaire for Stroke Caregivers) to assess the competence of family caregivers. The random-effects model in Figure 2(e) shows that the caregiving competence in the telerehabilitation group is significantly higher than that in the routine rehabilitation group (SMD = 1.35, 95% CI = 0.82∼1.88, *P* < 0.001, *I*^2^ = 65%). Different measurement tools may be one of the potential sources of high heterogeneity.

#### 3.4.6. Self-Efficacy

Four studies applied three scales (Self-efficacy Scale, Caregiving Self-efficacy Score, and General Self-efficacy Scale) to assess anxiety symptoms. The random-effects model in Figure 2(f) shows that the telerehabilitation group has no significant effect on self-efficacy to the routine rehabilitation group (SMD = −0.30, 95% CI = −1.22∼0.61, *P*=0.52, *I*^2^ = 89%). The high heterogeneity may result from different measurement tools.

## 4. Discussion

Previous studies paid more attention to the effects of telerehabilitation on stroke survivors. This is the first systematic review and meta-analysis focused on the impact of telerehabilitation on the health outcomes of stroke caregivers. Although some published systematic reviews attempted to compare the outcomes of stroke caregivers receiving telerehabilitation, they only performed descriptive analyses without further meta-analysis [33, 34]. This study pooled 16 studies and found that 3/4 of the studies implement telerehabilitation through websites and software, which demonstrated that more and more medical institutions tend to cooperate with Internet companies to build new platforms to realize medical modernization and intelligence. Stroke patients and caregivers can enjoy high-quality medical and nursing services without leaving home. Only RCTs were included in this study to ensure study quality and accuracy. However, 87.5% of the RCTs had some risk of bias due to poor design and missing data. The total sample size of the studies reporting anxiety, knowledge, competence, and self-efficacy was insufficient, resulting in an inferior quality of synthesized evidence for the four outcomes. Although the quality evaluation was rigorous, this study yielded some surprising results.

Moderate-quality evidence indicated that telerehabilitation can ease the burden and reduce the strain on stroke caregivers. A previous systematic review found that the caregiver strain index of stroke caregivers was comparable, which was consistent with the results of this study [34]. A qualitative study found that caregivers of stroke survivors were experiencing a stressful period [35]. New responsibilities leave family caregivers feeling resentful and guilty of self-sacrifice. Meanwhile, outpatient follow-up is stressful for stroke caregivers due to scheduling, transportation, and expenses. Telerehabilitation improved the feasibility and acceptability of the interventions. In Lelaurin's study, 80% of stroke caregivers considered telerehabilitation very or even extremely useful [26]. However, the majority of caregivers receiving telerehabilitation interventions have a high level of education. For caregivers with low education levels and poor network resources, receiving telerehabilitation makes them feel burdensome instead. This problem is also a major problem that needs to be overcome when implementing telerehabilitation.

Very low-quality evidence suggested that family caregivers have a substantial demand for caregiving knowledge and skills, which can be solved by telerehabilitation. An evidence-based guideline for stroke caregivers recommended websites as critical platforms to provide caregivers with information about stroke treatment and prognosis (level 3) [36]. Zhao's study suggested that the low competence of stroke caregivers is due to a lack of knowledge and skills [20]. Almost all 16 RCTs in this study mentioned the importance of improving the knowledge and skills of stroke caregivers, but only four assessed the results specifically. Thus, it is necessary to assess the knowledge and skills of stroke caregivers in various ways. After all, the knowledge and skills of caregivers directly affect the effects of home rehabilitation in patients with stroke.

Multiform telerehabilitation can improve caregiving competence. However, the effects of multiform telerehabilitation on depression were not significantly different from those of routine rehabilitation. Multiform telerehabilitation mainly provides interventions through at least two media to ensure patients and caregivers master knowledge and skills. This phenomenon indicates that multiform telerehabilitation has higher requirements for network equipment configuration than single-form telerehabilitation and routine rehabilitation. Unfortunately, no RCT has yet proven the hypothesis that multiform telerehabilitation may cause a waste of resources and increase the economic burden. In addition, more RCTs with direct and indirect comparisons between multiform telerehabilitation and single-form telerehabilitation are needed. In the near future, a network meta-analysis of multiform telerehabilitation, single-form telerehabilitation, and routine rehabilitation may be necessary.

The emotional state of stroke caregivers deserves attention, but it is often ignored. The current study cannot demonstrate the effectiveness of telerehabilitation in improving anxiety, depression, and self-efficacy. This finding did not mean that telerehabilitation cannot affect caregivers' emotions. Due to the inconsistent duration of intervention, caregivers' anxiety, depression, and self-efficacy may require long-term observation. Besides, limited studies also lead to nonstatistically significant differences. At present, telerehabilitation mainly provides caregiving knowledge and skills to help stroke survivors get better care and indirectly relieve the anxiety and depression of family caregivers. There is a lack of direct telerehabilitation interventions for caregivers' anxiety and depression symptoms. Healthcare providers should pay close attention to the psychological and emotional changes of stroke caregivers for a long time and take timely treatment measures.

The prospect of telerehabilitation for family caregivers of stroke survivors is broad and promising. Nevertheless, some challenges come along (1) the education level and digital literacy of stroke caregivers need to be taken into account while implementing telerehabilitation; (2) healthcare facilities need to complete the mechanism of telerehabilitation supervision to ensure the safety and effectiveness; (3) remote consultation and therapies need to be provided based on caregivers' physical and psychological stress. Most of the family caregivers in this study were aged between 25 and 84, indicating that the design of telerehabilitation should be more accessible and meet the personalized needs of different age groups.

Several limitations should be acknowledged in this systematic review. Firstly, most of the telerehabilitation programs were offered to stroke survivors and their family caregivers simultaneously, leading to the possibility of some interaction effects. Secondly, although we used SMD to combine the effect size, different measurement tools may bring potential heterogeneity. Finally, due to limited studies, subgroup analysis and sensitivity analysis were only performed in caregiver burden and depression. Thus, more high-quality RCTs should be carried out to support our results.

## 5. Conclusion

Providing telerehabilitation for family caregivers of stroke survivors can improve caregiver burden, knowledge, and competence. Multiform telerehabilitation plays a positive role in improving caregiving competence. High-quality studies and individualized telerehabilitation are needed to be tailed for family caregivers of stroke survivors.

## 6. Implications for Nursing Management

After stroke survivors are discharged from the hospital, their family caregivers typically take on the main caregiving role. Sudden caregiving task makes stroke caregivers more vulnerable to physical and mental stress, which is often neglected by medical staff. The emergence of telerehabilitation can help relieve caregivers' stress and improve their adverse outcomes. In addition, telerehabilitation provides a new form for nursing managers to make discharge plans for stroke.

## Figures and Tables

**Table 1:** The characteristics of included studies.

Author, year	Country	Sample size (EG/CG)	Age of patients (EG/CG)	Age of caregiver (EG/CG)	Interventions (EG/CG)	Intervention format	Intervention duration	Outcomes
			(Mean ± SD)	(Mean ± SD)				
Radomski, 2007 [18]	USA	EG: *n* = 5	47–72/24–81	—	EG: habit training via a wireless device during a four- to five-week practice period	Web-based software	5 weeks	Caregiver burden
		CG: *n* = 5			CG: usual and customary practice (received no therapeutic intervention during the home visits)			

Pierce et al., 2009 [17]	USA	EG: *n* = 36	63 ± 15/63 ± 13.3	54 ± 12.2/55 ± 13.1	EG: caring-web intervention: (1) linked websites about stroke care; (2) customized educational information to carers' needs; (3) an e-mail forum to ask any questions in private; and (4) a nonstructured e-mail discussion amongst all participants facilitated by the nurse	Website + e-mail	12 months	Depression
		CG: *n* = 37			CG: non-web user group			

Kim and Park, 2011 [16]	Korea	EG: *n* = 17	59.6 ± 9.5/58.5 ± 8.8	38.3 ± 12.6/42.9 ± 12.0	EG: web-based stroke prevention education program	Website	12 weeks	Caregiving knowledge
		CG: *n* = 17			CG: usual care			

Harwood et al., 2012 [25]	New Zealand	EG: *n* = 39	61.5 ± 13.9/61.3 ± 14.8	—	EG: DVD and take charge session. 80-minute DVD about recovery and 80-minute individualized assessment	DVD	12 months	Caregiver burden
		CG: *n* = 39			CG: written materials about stroke for patients and their families			

Redzuan et al., 2012 [10]	Malaysia	EG: *n* = 44	63.7 ± 12/59.4 ± 11	—	EG: received a video to guide therapy at home. The video consisted of 6 sections and included a 45-minute self-instructional therapy in 2 languages	DVD	3 months	Caregiver burden
		CG: *n* = 46			CG: verbal instruction and outpatient appointments once a week			

Eames et al., 2013 [27]	Australia	EG: *n* = 31	61.4 ± 12.7/55.2 ± 16.7	61.4 ± 12.7/55.2 ± 16.7	EG: stroke education and support packages were applied to patients and caregivers. Telephone sessions at intervals of approximately 1 month, over a 3-month period	Website + telephone sessions	3 months	Caregiver burden; anxiety; depression; self-efficacy; caregiving knowledge
		CG: *n* = 30			CG: standard care			

Kim et al., 2013 [28]	Korea	EG: *n* = 18	67.4 ± 7.3/63.9 ± 7.4	49.8 ± 14.8)/57.3 ± 11.5	EG: the web-based stroke education program included nine video-based lectures within 9 weeks	Website	9 weeks	Caregiving competence
		CG: *n* = 18			CG: standard care			

Blanton, 2016 [24]	USA	EG: *n* = 21	56.5 ± 12.6)/60.1 ± 14.0	56.5 ± 12.6)/60.1 ± 14.0	EG: CARE-CITE education program carepartners (caregivers participated in online CARE-CITE education while stroke survivors received constraint-induced movement therapy)	Website	1 month	Caregiver burden; depression; self-efficacy
		CG: *n* = 11			CG: traditional education carepartners (carepartners participated in traditional education while the stroke survivor received constraint-induced movement therapy)			

Berg et al., 2016 [19]	Australia	EG: *n* = 31	64.5 ± 18.5/70.1 ± 12.4	—	EG: an 8-week caregiver-mediated training program with support using a customized exercise app loaded onto a tablet	Web-based software + video conferencing	8 weeks	Caregiver burden; self-efficacy
		CG: *n* = 32			CG: usual rehabilitation care			

Chen et al., 2017 [29]	China	EG: *n* = 27	66.52 ± 12.08/66.15 ± 12.33	—	EG: the home telesupervising rehabilitation program consisted of a network data system, therapist end, and patient end	Web-based software	24 weeks	Caregiver burden
		CG: *n* = 27			CG: conventional outpatient rehabilitation			

Cheng et al., 2018 [30]	Hong Kong	EG: *n* = 64	71.16 ± 11.89/70.59 ± 10.36	49.08 ± 12.09/49.11 ± 12.90	EG: the psychoeducational program is composed of (1) two structured individual face-to-face stroke education sessions; and (2) six biweekly telephone problem-solving skills training sessions. An information booklet was developed as reference material for caregivers	Telephone sessions	26 weeks	Caregiver burden; depression; caregiving competence
		CG: *n* = 64			CG: routine care (interdisciplinary rehabilitation services and postdischarge outpatient medical follow-up)			

Kootker et al., 2019 [31]	Netherlands	EG: *n* = 23	≥18	59.6 ± 10.2/59.8 ± 12.5	EG: computerized cognitive training with 13–16 sessions. Each session will take two 20–25 minute-blocks divided by a 10–15 minute-break	Web-based software	4 months	Caregiver burden; anxiety; depression
		CG: *n* = 27			CG: cognitive behavioral therapy augmented with movement or occupational therapy			

Vloothuis et al., 2019 [32]	Netherlands	EG: *n* = 32	60.53 ± 14.82/59.26 ± 15.01	53.91 ± 14.90/54.00 ± 12.26	EG: caregiver-mediated exercises at least five times a week for 30 minutes	Telephone session + video conferencing + e-mail	8 weeks	Caregiver burden; anxiety; depression; self-efficacy
		CG: *n* = 34			CG: usual care			

LeLaurin et al., 2021 [26]	USA	EG: *n* = 13	70.6 ± 10.7	60.3 ± 10.1	EG: eight-week intervention (the intervention was based on the RESCUE website and delivered via telephone in 8 weekly sessions lasting 30 to 60 minutes each)	Website + telephone sessions	8 weeks	Caregiver burden; depression
		CG: *n* = 14			CG: standard care			

Woodward et al., 2021 [23]	USA	EG: *n* = 54	≥18	69.8 ± 13.8/58.1 ± 15.5	EG: SWCM + MISTT website (the MISTT website included a range of resources and stroke-related information)	Website	60 days	Depression
		CG: *n* = 58			CG: usual care (the health-related brochures were mailed at 1, 4, and 8 week postdischarge)			

Zhao et al., 2021 [20]	China	EG: *n* = 38	62.50 ± 12.02/62.95 ± 10.21	43.87 ± 11.35/44.54 ± 11.56	EG: Internet combined with information, motivation, and behavior model	Web-based software + WeChat	5 weeks	Caregiver burden; caregiving competence; caregiving knowledge
		CG: *n* = 39			CG: usual care			

*Note.* EG, experimental group; CG, control group; SD, standard deviation; SWCM, social work case management; MISTT, Michigan stroke transitions trial.

## Data Availability

The data supporting this study are from previously reported studies and datasets, which have already been cited.

## Supplementary Materials

Supplementary Materials Supplementary Table. 1. Search strategy. Supplementary Figure 1. Risk of bias graph. Supplementary Figure 2. Subgroup analysis. Supplementary Table. 2. Overall evidence quality of each outcome.
