# Supplementary material for: Telerehabilitation for Family Caregivers of Stroke Survivors: A Systematic Review and Meta-Analysis
Source: J Nurs Manag. 2023 Jun 14;2023:3450312. doi: 10.1155/2023/3450312 (PMC11918953; doi:10.1155/2023/3450312)
Supplement: Supplementary Materials — Supplementary Table. 1. Search strategy. Supplementary Figure 1. Risk of bias graph. Supplementary Figure 2. Subgroup analysis. Supplementary Table. 2. Overall evidence quality of each outcome. [file 3450312.f1.doc]

*Supplementary Materials*

**Supplementary Table. 1. Search strategy 2**

**Supplementary Fig.1. Risk of bias graph 8**

**Supplementary Table. 2. Overall evidence quality of each outcome 9**

**Supplementary Fig.2. Subgroup analysis. 11**

1. Caregiver burden 11
2. Depression 11

**Supplementary Table. 1. Search strategy**

| **Database** | **No** | **Search strategy** |
| --- | --- | --- |
| PubMed | #1 | "Stroke"[MeSH Terms] OR "Cerebrovascular Disorders"[MeSH Terms] OR "Basal Ganglia Cerebrovascular Disease"[MeSH Terms] OR "Brain Ischemia"[MeSH Terms] OR "Intracranial Hemorrhages"[MeSH Terms] OR "Intracranial Embolism and Thrombosis"[MeSH Terms] OR "Intracranial Arterial Diseases"[MeSH Terms] OR "Cerebral Small Vessel Diseases"[MeSH Terms] |
|  | #2 | (((((((((((Stroke[Title/Abstract]) OR (Cerebrovascular Disorders[Title/Abstract])) OR (Basal Ganglia Cerebrovascular Disease[Title/Abstract])) OR (brain ischemia[Title/Abstract])) OR (Intracranial Hemorrhages[Title/Abstract])) OR (Intracranial Embolism[Title/Abstract] AND Thrombosis[Title/Abstract])) OR (Intracranial Arterial Diseases[Title/Abstract])) OR (Cerebral Small Vessel Diseases[Title/Abstract])) OR (cerebrovasc*[Title/Abstract])) OR (brain vasc*[Title/Abstract])) OR (cerebral vasc*[Title/Abstract])) OR (apoplex*[Title/Abstract]) |
|  | #3 | (brain*[Title/Abstract] OR cerebr*[Title/Abstract] OR cerebell*[Title/Abstract] OR vertebrobasilar[Title/Abstract] OR hemispher*[Title/Abstract] OR intracran*[Title/Abstract] OR intracerebral[Title/Abstract] OR infratentorial[Title/Abstract] OR supratentorial[Title/Abstract] OR MCA[Title/Abstract] OR anterior circulation[Title/Abstract] OR posterior circulation[Title/Abstract] OR basal ganglia[Title/Abstract]) AND (isch*emi*[Title/Abstract] OR infarct*[Title/Abstract] OR thrombo*[Title/Abstract] OR emboli*[Title/Abstract]) |
|  | #4 | ((((((((((brain*[Title/Abstract]) OR (cerebr*[Title/Abstract])) OR (cerebell*[Title/Abstract])) OR (intracerebral[Title/Abstract])) OR (intracran*[Title/Abstract])) OR (parenchymal[Title/Abstract])) OR (intraventricular[Title/Abstract])) OR (infratentorial[Title/Abstract])) OR (supratentorial[Title/Abstract])) OR (basal gangli*[Title/Abstract])) AND (((((haemorrhage*[Title/Abstract]) OR (hemorrhage*[Title/Abstract])) OR (haematoma*[Title/Abstract])) OR (hematoma*[Title/Abstract])) OR (bleed*[Title/Abstract])) |
|  | #5 | #1 OR #2 OR #3 OR #4 |
|  | #6 | ((((("Caregivers"[Mesh]) OR "Caregiver Burden"[Mesh]) OR "Family"[Mesh]) OR "Family Therapy"[Mesh]) OR "Family Health"[Mesh]) OR "Family Characteristics"[Mesh] |
|  | #7 | (((((((((((((((((((((((Caregivers[Title/Abstract]) OR (Caregiver Burden[Title/Abstract])) OR (Family[Title/Abstract])) OR (Family Therapy[Title/Abstract])) OR (Family Health[Title/Abstract])) OR (family Characteristics[Title/Abstract])) OR (family member*[Title/Abstract])) OR (Family relation*[Title/Abstract])) OR (Carer*[Title/Abstract])) OR (Care giver*[Title/Abstract])) OR (Care-giver*[Title/Abstract])) OR (Families[Title/Abstract])) OR (Parent?[Title/Abstract])) OR (Partner*[Title/Abstract])) OR (Father[Title/Abstract])) OR (Mather[Title/Abstract])) OR (Husband[Title/Abstract])) OR (Wife[Title/Abstract])) OR (Wives[Title/Abstract])) OR (Child*[Title/Abstract])) OR (Spouse*[Title/Abstract])) OR (Friend*[Title/Abstract])) OR (Relative*[Title/Abstract])) OR (Home health aside*[Title/Abstract]) |
|  | #8 | #6 OR #7 |
|  | #9 | ((("Telemedicine"[Mesh]) OR "Telecommunications"[Mesh]) OR "Mobile Applications"[Mesh]) OR "Computer Communication Networks"[Mesh] |
|  | #10 | (((((((((((((((((((((((Tele*[Title/Abstract]) OR (computer communication networks[Title/Abstract])) OR (mobile application[Title/Abstract])) OR (Video*[Title/Abstract])) OR (eHealth[Title/Abstract])) OR (E-Health[Title/Abstract])) OR (Electronic health[Title/Abstract])) OR (Mail[Title/Abstract])) OR (email[Title/Abstract])) OR (e-mail[Title/Abstract])) OR (Internet*[Title/Abstract])) OR (Remote[Title/Abstract])) OR (Phone[Title/Abstract])) OR (Web[Title/Abstract])) OR (Smartphone[Title/Abstract])) OR (text‐messag*[Title/Abstract])) OR (Mhealth[Title/Abstract])) OR (m‐health[Title/Abstract])) OR (mobile health[Title/Abstract])) OR (Pedometer*[Title/Abstract])) OR (Actigraph*[Title/Abstract])) OR (Acceleromet*[Title/Abstract])) OR (activity tracker[Title/Abstract])) OR (app[Title/Abstract]) |
|  | #11 | (remote[Title/Abstract] OR distance[Title/Abstract] OR distant[Title/Abstract]) AND (rehabilitation[Title/Abstract] OR therap*[Title/Abstract] OR treatment[Title/Abstract] OR physio*[Title/Abstract] OR communication[Title/Abstract] OR consultation[Title/Abstract] OR care[Title/Abstract] OR specialist*[Title/Abstract] OR monitor[Title/Abstract] OR virtual reality[Title/Abstract] OR VR[Title/Abstract] OR virtual environment[Title/Abstract] OR technolog*[Title/Abstract]) |
|  | #12 | (physical[Title/Abstract] OR physiolog*[Title/Abstract] OR perform*[Title/Abstract] OR train*[Title/Abstract] OR activ*[Title/Abstract] OR endur*[Title/Abstract] OR exercise*[Title/Abstract]) AND (track*[Title/Abstract] OR monitor*[Title/Abstract] OR measur*[Title/Abstract] OR device*[Title/Abstract] OR app[Title/Abstract]) |
|  | #13 | (step*[Title/Abstract] OR walk*[Title/Abstract]) AND (count*[Title/Abstract] OR meter*[Title/Abstract] OR daily[Title/Abstract]) |
|  | #14 | #9 OR #10 OR #11 OR #12 OR #13 |
|  | #15 | (((randomized controlled trial[Publication Type]) OR (randomized[Title/Abstract])) OR (randomised[Title/Abstract])) OR (placebo[Title/Abstract]) |
|  | #16 | #5 AND #8 AND #14 AND #15 |
| **Database** | **No** | **Search strategy** |
| Embase | #1 | 'cerebrovascular disease'/mj OR 'basal ganglion hemorrhage'/exp OR 'cerebrovascular accident'/exp OR 'brain hemangioma'/exp OR 'brain hematoma'/exp OR 'brain infarction'/exp OR 'brain ischemia'/exp OR 'cerebral artery disease'/exp OR 'cerebrovascular accident'/exp OR 'hypophysis apoplexy'/exp OR 'occlusive cerebrovascular disease'/exp OR 'vertebrobasilar insufficiency'/exp OR 'brain hemorrhage'/exp |
|  | #2 | 'cerebrovascular disease':ti,ab,kw OR 'basal ganglion hemorrhage':ti,ab,kw OR 'brain hemangioma':ti,ab,kw OR 'brain hematoma':ti,ab,kw OR 'brain infarction':ti,ab,kw OR 'brain ischemia':ti,ab,kw OR 'cerebral artery disease':ti,ab,kw OR 'cerebrovascular accident':ti,ab,kw OR 'hypophysis apoplexy':ti,ab,kw OR 'occlusive cerebrovascular disease':ti,ab,kw OR 'vertebrobasilar insufficiency':ti,ab,kw OR 'brain hemorrhage':ti,ab,kw OR stroke:ti,ab,kw OR 'cerebrovascular disorders':ti,ab,kw OR 'intracranial hemorrhages':ti,ab,kw OR ('intracranial embolism':ti,ab,kw AND thrombosis:ti,ab,kw) OR 'intracranial arterial diseases':ti,ab,kw OR 'cerebral small vessel diseases':ti,ab,kw OR cerebrovasc*:ti,ab,kw OR 'brain vasc*':ti,ab,kw OR 'cerebral vasc*':ti,ab,kw OR apoplex*:ti,ab,kw |
|  | #3 | (brain*:ti,ab,kw OR cerebr*:ti,ab,kw OR cerebell*:ti,ab,kw OR vertebrobasilar:ti,ab,kw OR hemispher*:ti,ab,kw OR intracran*:ti,ab,kw OR intracerebral:ti,ab,kw OR infratentorial:ti,ab,kw OR supratentorial:ti,ab,kw OR mca:ti,ab,kw OR 'anterior circulation':ti,ab,kw OR 'posterior circulation':ti,ab,kw OR 'basal ganglia':ti,ab,kw) AND (isch*emi*:ti,ab,kw OR infarct*:ti,ab,kw OR thrombo*:ti,ab,kw OR emboli*:ti,ab,kw) |
|  | #4 | (brain*:ti,ab,kw OR cerebr*:ti,ab,kw OR cerebell*:ti,ab,kw OR intracerebral:ti,ab,kw OR intracran*:ti,ab,kw OR parenchymal:ti,ab,kw OR intraventricular:ti,ab,kw OR infratentorial:ti,ab,kw OR supratentorial:ti,ab,kw OR 'basal gangli*':ti,ab,kw) AND (haemorrhage*:ti,ab,kw OR hemorrhage*:ti,ab,kw OR haematoma*:ti,ab,kw OR hematoma*:ti,ab,kw OR bleed*:ti,ab,kw) |
|  | #5 | #1 OR #2 OR #3 OR #4 |
|  | #6 | 'caregiver'/exp OR 'caregiver burden'/exp OR 'caregiver support'/exp OR 'family'/exp OR 'family therapy'/exp OR 'parent'/exp OR 'relative'/exp OR 'family centered care'/exp |
|  | #7 | caregiver*:ti,ab,kw OR 'caregiver burden':ti,ab,kw OR 'caregiver support':ti,ab,kw OR family:ti,ab,kw OR 'family therapy':ti,ab,kw OR parent*:ti,ab,kw OR 'family centered care':ti,ab,kw OR 'family health':ti,ab,kw OR 'family characteristics':ti,ab,kw OR 'family member*':ti,ab,kw OR 'family relation*':ti,ab,kw OR carer*:ti,ab,kw OR 'care giver*':ti,ab,kw OR families:ti,ab,kw OR partner*:ti,ab,kw OR father:ti,ab,kw OR mother:ti,ab,kw OR child*:ti,ab,kw OR husband:ti,ab,kw OR wife:ti,ab,kw OR wives:ti,ab,kw OR spouse*:ti,ab,kw OR friend*:ti,ab,kw OR relative*:ti,ab,kw OR 'home health aside*':ti,ab,kw |
|  | #8 | #6 OR #7 |
|  | #9 | 'telehealth'/exp OR 'telemedicine'/exp OR 'telephone'/exp OR 'telecommunication'/exp OR 'mobile application'/exp OR 'computer network'/exp OR 'videoconferencing'/exp OR 'videorecording'/exp |
|  | #10 | tele*:ti,ab,kw OR 'computer communication networks':ti,ab,kw OR 'mobile application':ti,ab,kw OR video*:ti,ab,kw OR ehealth:ti,ab,kw OR 'e health':ti,ab,kw OR 'electronic health':ti,ab,kw OR mail:ti,ab,kw OR email:ti,ab,kw OR 'e mail':ti,ab,kw OR internet*:ti,ab,kw OR remote:ti,ab,kw OR phone:ti,ab,kw OR web:ti,ab,kw OR smartphone:ti,ab,kw OR text‐messag*:ti,ab,kw OR mhealth:ti,ab,kw OR m‐health:ti,ab,kw OR 'mobile health':ti,ab,kw OR pedometer*:ti,ab,kw OR actigraph*:ti,ab,kw OR acceleromet*:ti,ab,kw OR 'activity tracker':ti,ab,kw OR app:ti,ab,kw OR apps:ti,ab,kw OR 'computer network':ti,ab,kw OR 'wireless communication':ti,ab,kw |
|  | #11 | (remote:ti,ab,kw OR distance:ti,ab,kw OR distant:ti,ab,kw) AND (rehabilitation:ti,ab,kw OR therap*:ti,ab,kw OR treatment:ti,ab,kw OR physio*:ti,ab,kw OR communication:ti,ab,kw OR consultation:ti,ab,kw OR care:ti,ab,kw OR specialist*:ti,ab,kw OR monitor:ti,ab,kw OR 'virtual reality':ti,ab,kw OR vr:ti,ab,kw OR 'virtual environment':ti,ab,kw OR technolog*:ti,ab,kw) |
|  | #12 | (physical:ti,ab,kw OR physiolog*:ti,ab,kw OR perform*:ti,ab,kw OR train*:ti,ab,kw OR activ*:ti,ab,kw OR endur*:ti,ab,kw OR exercise*:ti,ab,kw) AND (track*:ti,ab,kw OR monitor*:ti,ab,kw OR measur*:ti,ab,kw OR device*:ti,ab,kw OR app:ti,ab,kw OR apps:ti,ab,kw) |
|  | #13 | (step*:ti,ab,kw OR walk*:ti,ab,kw) AND (count*:ti,ab,kw OR meter*:ti,ab,kw OR daily:ti,ab,kw) |
|  | #14 | #9 OR #10 OR #11 OR #12 OR #13 |
|  | #15 | random* OR placebo:it OR 'double blind' |
|  | #16 | #5 AND #8 AND #14 AND #15 |
| **Database** | **No** | **Search strategy** |
| Cochrane Library | #1 | MeSH descriptor: [Stroke] explode all trees |
|  | #2 | MeSH descriptor: [Cerebrovascular Disorders] explode all trees |
|  | #3 | MeSH descriptor: [Basal Ganglia Cerebrovascular Disease] explode all trees |
|  | #4 | MeSH descriptor: [Brain Ischemia] explode all trees |
|  | #5 | MeSH descriptor: [Intracranial Hemorrhages] explode all trees |
|  | #6 | MeSH descriptor: [Intracranial Embolism and Thrombosis] explode all trees |
|  | #7 | MeSH descriptor: [Intracranial Arterial Diseases] explode all trees |
|  | #8 | #1 OR #2 OR #3 OR #4 OR #5 OR #6 OR #7 |
|  | #9 | (Stroke OR Cerebrovascular Disorders OR Basal Ganglia Cerebrovascular Disease OR brain ischemia OR Intracranial Hemorrhages OR (Intracranial Embolism And Thrombosis) OR Intracranial Arterial Diseases OR Cerebral Small Vessel Diseases OR cerebrovasc* OR brain vasc* OR cerebral vasc* OR apoplex*):ti,ab,kw |
|  | #10 | ((brain* OR cerebr* OR cerebell* OR vertebrobasilar OR hemispher* OR intracran* OR intracerebral OR infratentorial OR supratentorial OR MCA OR anterior circulation OR posterior circulation OR basal ganglia) AND (isch*emi* OR infarct* OR thrombo* OR emboli*)):ti,ab,kw |
|  | #11 | ((brain* OR cerebr* OR cerebell* OR intracerebral OR intracran* OR parenchymal OR intraventricular OR infratentorial OR supratentorial OR basal gangli*) AND (haemorrhage* OR hemorrhage* OR haematoma* OR hematoma* OR bleed*)):ti,ab,kw |
|  | #12 | #8 OR #9 OR #10 OR #11 |
|  | #13 | MeSH descriptor: [Caregivers] explode all trees |
|  | #14 | MeSH descriptor: [Caregiver Burden] explode all trees |
|  | #15 | MeSH descriptor: [Family] explode all trees |
|  | #16 | MeSH descriptor: [Family Therapy] explode all trees |
|  | #17 | MeSH descriptor: [Family Health] explode all trees |
|  | #18 | MeSH descriptor: [Family Characteristics] explode all trees |
|  | #19 | #13 OR #14 OR #15 OR #16 OR #17 OR #18 |
|  | #20 | (Caregivers OR Caregiver Burden OR Family OR Family Therapy OR Family Health OR family Characteristics OR family member* OR Family relation* OR Carer* OR Care giver* OR Care-giver* OR Families OR Parent? OR Partner* OR Father OR Mather OR Husband OR Wife OR Wives OR Child* OR Spouse* OR Friend* OR Relative* OR Home health aside*):ti,ab,kw |
|  | #21 | #19 OR #20 |
|  | #22 | MeSH descriptor: [Telemedicine] explode all trees |
|  | #23 | MeSH descriptor: [Telecommunications] explode all trees |
|  | #24 | MeSH descriptor: [Mobile Applications] explode all trees |
|  | #25 | MeSH descriptor: [Computer Communication Networks] explode all trees |
|  | #26 | #22 OR #23 OR #24 OR #25 |
|  | #27 | (Tele* OR computer communication networks OR mobile application OR Video* OR eHealth OR E-Health OR Electronic health OR Mail OR email OR e-mail OR Internet* OR Remote OR Phone OR Web OR Smartphone OR text‐messag* OR Mhealth OR m‐health OR mobile health OR Pedometer* OR Actigraph* OR Acceleromet* OR activity tracker OR app):ti,ab,kw |
|  | #28 | ((remote OR distance OR distant) AND (rehabilitation OR therap* OR treatment OR physio* OR communication OR consultation OR care OR specialist* OR monitor OR virtual reality OR VR OR virtual environment OR technolog*)):ti,ab,kw |
|  | #29 | ((physical OR physiolog* OR perform* OR train* OR activ* OR endur* OR exercise*) AND (track* OR monitor* OR measur* OR device* OR app)):ti,ab,kw |
|  | #30 | ((step* OR walk*) AND (count* OR meter* OR daily)):ti,ab,kw |
|  | #31 | #26 OR #27 OR #28 OR #29 OR #30 |
|  | #32 | (random* OR placebo OR double blind):ti,ab,kw |
|  | #33 | #12 AND #21 AND #31 AND #32 |
| **Database** | **No** | **Search strategy** |
| Web of Science | #1 | TS=(Stroke OR Cerebrovascular Disorders OR Basal Ganglia Cerebrovascular Disease OR brain ischemia OR Intracranial Hemorrhages OR (Intracranial Embolism And Thrombosis) OR Intracranial Arterial Diseases OR Cerebral Small Vessel Diseases OR cerebrovasc* OR brain vasc* OR cerebral vasc* OR apoplex*) |
|  | #2 | TS=((brain* OR cerebr* OR cerebell* OR vertebrobasilar OR hemispher* OR intracran* OR intracerebral OR infratentorial OR supratentorial OR MCA OR anterior circulation OR posterior circulation OR basal ganglia) AND (isch*emi* OR infarct* OR thrombo* OR emboli*) ) |
|  | #3 | TS=((brain* OR cerebr* OR cerebell* OR intracerebral OR intracran* OR parenchymal OR intraventricular OR infratentorial OR supratentorial OR basal gangli*) AND (haemorrhage* OR hemorrhage* OR haematoma* OR hematoma* OR bleed*)) |
|  | #4 | #1 OR #2 OR #3 |
|  | #5 | TS=(Caregivers OR Caregiver Burden OR Family OR Family Therapy OR Family Health OR family Characteristics OR family member* OR Family relation* OR Carer* OR Care giver* OR Care-giver* OR Families OR Parent? OR Partner* OR Father OR Mather OR Husband OR Wife OR Wives OR Child* OR Spouse* OR Friend* OR Relative* OR Home health aside*) |
|  | #6 | **TS=(Tele* OR computer communication networks OR mobile application OR Video* OR eHealth OR E-Health OR Electronic health OR Mail OR email OR e-mail OR Internet* OR Remote OR Phone OR Web OR Smartphone OR text‐messag* OR Mhealth OR m‐health OR mobile health OR Pedometer* OR Actigraph* OR Acceleromet* OR activity tracker OR app)** |
|  | #7 | **TS=((remote OR distance OR distant) AND (rehabilitation OR therap* OR treatment OR physio* OR communication OR consultation OR care OR specialist* OR monitor OR virtual reality OR VR OR virtual environment OR technolog*))** |
|  | #8 | **TS=((physical OR physiolog* OR perform* OR train* OR activ* OR endur* OR exercise*) AND (track* OR monitor* OR measur* OR device* OR app))** |
|  | #9 | **TS=((step* OR walk*) AND (count* OR meter* OR daily))** |
|  | #10 | **#6 OR #7 OR #8 OR #9** |
|  | #11 | **TS=(random* OR placebo OR double blind)** |
|  | #12 | **#4 AND #5 AND #10 AND #11** |
| **Database** | **No** | **Search strategy** |
| CINAHL | S1 | (MH "Stroke+") OR (MH "Cerebrovascular Disorders+") OR (MH "Basal Ganglia Cerebrovascular Disease+") OR (MH "Cerebral Ischemia+") OR (MH "Intracranial Hemorrhage+") OR (MH "Intracranial Embolism and Thrombosis+") OR (MH "Intracranial Arterial Diseases+") OR (MH "Cerebral Small Vessel Diseases+") |
|  | S2 | (MM "Caregivers") OR (MM "Caregiver Burden") OR (MM "Caregiver Support") OR (MH "Family+") OR (MM "Family Therapy") OR (MM "Family Health") OR (MH "Family Characteristics+") |
|  | S3 | (MH "Telemedicine+") OR (MM "Telerehabilitation") OR (MH "Telehealth+") OR (MH "Telecommunications+") OR (MM "Mobile Applications") OR (MH "Computer Communication Networks+") |
|  | S4 | TX Tele* OR computer communication networks OR mobile application OR Video* OR eHealth OR E-Health OR Electronic health OR Mail OR email OR e-mail OR Internet* OR Remote OR Phone OR Web OR Smartphone OR text‐messag* OR Mhealth OR m‐health OR mobile health OR Pedometer* OR Actigraph* OR Acceleromet* OR activity tracker OR app |
|  | S5 | TX ( remote OR distance OR distant ) AND TX ( rehabilitation OR therap* OR treatment OR physio* OR communication OR consultation OR care OR specialist* OR monitor OR virtual reality OR VR OR virtual environment OR technolog* ) |
|  | S6 | TX ( physical OR physiolog* OR perform* OR train* OR activ* OR endur* OR exercise* ) AND TX ( track* OR monitor* OR measur* OR device* OR app ) |
|  | S7 | TX ( step* OR walk* ) AND TX ( count* OR meter* OR daily ) |
|  | S8 | S3 OR S4 OR S5 OR S6 OR S7 |
|  | S9 | TX random* OR placebo OR double blind |
|  | S10 | S1 OR S2 OR S8 OR S9 |
| **Database** | **No** | **Search strategy** |
| PsycINFO | S1 | ( MM "Cerebrovascular Accidents" OR DE "Cerebrovascular Disorders" OR DE "Cerebral Arteriosclerosis" OR DE "Cerebral Hemorrhage" OR DE "Cerebral Ischemia" OR DE "Cerebral Small Vessel Disease" OR DE "Cerebrovascular Accidents" OR DE "Subarachnoid Hemorrhage" ) OR TX ( stroke OR cerebrovascular disorder* OR brain ischemia OR Intracranial Hemorrhage OR TIA OR transient ischemia attack* OR cerebral hemorrhage OR cerebral Ischemia ) |
|  | S2 | ((MM "Caregiver Burden" OR MM "Caregivers" OR MM "Caregiving") AND (DE "Family" OR DE "Biological Family" OR DE "Dual Careers" OR DE "Dysfunctional Family" OR DE "Extended Family" OR DE "Family Background" OR DE "Family History" OR DE "Family Members" OR DE "Family of Origin" OR DE "Family Relations" OR DE "Family Resemblance" OR DE "Family Structure" OR DE "Family Work Relationship" OR DE "Interethnic Family" OR DE "Interracial Family" OR DE "Military Families" OR DE "Nepotism" OR DE "Nuclear Family" OR DE "Schizophrenogenic Family" OR DE "Stepfamily" OR MM "Family Intervention" OR DE "Family Members" OR DE "Adopted Children" OR DE "Adult Offspring" OR DE "Ancestors" OR DE "Biological Family" OR DE "Cousins" OR DE "Daughters" OR DE "Foster Children" OR DE "Grandchildren" OR DE "Grandparents" OR DE "Illegitimate Children" OR DE "Inlaws" OR DE "Only Children" OR DE "Orphans" OR DE "Parents" OR DE "Siblings" OR DE "Sons" OR DE "Spouses" OR DE "Stepchildren") ) OR TX ( Caregiver* OR Family OR Carer* OR Care giver* OR Care-giver* OR Families OR Partner* ) |
|  | S3 | ((MM "Telerehabilitation") OR (MM "Telemedicine") ) OR TX ( telerehabilitation OR tele-rehabilitation OR virtual rehabilitation OR remote rehabilitation OR internet-based OR web-based OR mobile application OR eHealth OR E-Health OR Electronic health OR Internet* OR Phone OR Web OR Smartphone OR text‐messag* OR Mhealth OR m‐health OR mobile health OR app ) |
|  | S4 | TI ( random* OR placebo OR double blind ) OR AB ( random* OR placebo OR double blind ) OR KW ( random* OR placebo OR double blind ) |
|  | S5 | S1 AND S2 AND S3 AND S4 |
| **Total** |  |  |

**Supplementary Fig.1. Risk of bias graph.**

**
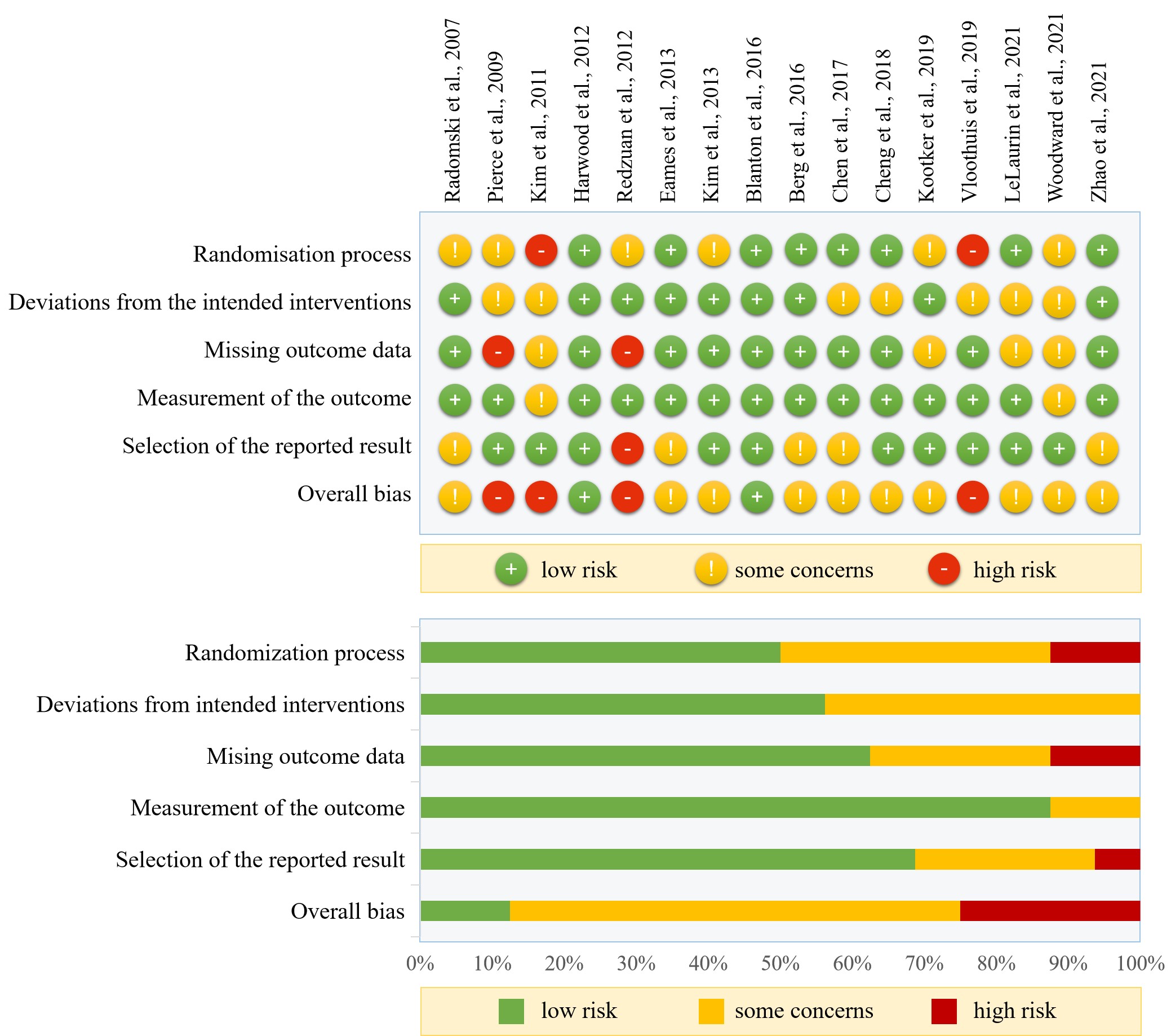
**

**Supplementary Table. 2. Overall evidence quality of each outcome**

| **Certainty assessment** | | | | | | | | **No. of patients** | | **Effect** | | | **Certainty** | **Importance** |
| --- | --- | --- | --- | --- | --- | --- | --- | --- | --- | --- | --- | --- | --- | --- |
| **No.** | **Study design** | **Risk of bias** | **Inconsistency** | **Indirectness** | **Imprecision** | **Other considerations** | **Telerabilitation** | | **Routine rehabilitation** | | **Relative (95% CI)** | **Absolute (95% CI)** |
| **Caregiver burden** | | | | | | | | | | | | | | |
| 11 | randomised trials | serious^a^ | not serious | not serious | not serious | none | 287 | | 274 | | - | SMD **0.18 lower** (0.35 lower to 0.02 lower) | ⨁⨁⨁◯ Moderate | CRITICAL |
| **Depression** | | | | | | | | | | | | | | |
| 8 | randomised trials | serious^a^ | not serious | not serious | not serious | none^b^ | 237 | | 229 | | - | SMD **0.04 lower** (0.3 lower to 0.21 higher) | ⨁⨁⨁◯ Moderate | CRITICAL |
| **Anxiety** | | | | | | | | | | | | | | |
| 3 | randomised trials | serious^c^ | not serious | not serious | serious^d^ | none^b^ | 69 | | 69 | | - | MD **0.68 higher** (0.68 lower to 2.04 higher) | ⨁⨁◯◯ Low | CRITICAL |
| **Caregiving knowledge** | | | | | | | | | | | | | | |
| 3 | randomised trials | serious^e^ | serious^f^ | not serious | serious^g^ | none^b^ | 80 | | 84 | | - | SMD **0.75 higher** (0.03 higher to 1.47 higher) | ⨁◯◯◯ Very low | CRITICAL |
| **Caregiving competence** | | | | | | | | | | | | | | |
| 3 | randomised trials | serious^a^ | serious^h^ | not serious | serious^g^ | none^b^ | 111 | | 106 | | - | SMD **1.35 higher** (0.82 higher to 1.88 higher) | ⨁◯◯◯ Very low | CRITICAL |
| **Self-efficacy** | | | | | | | | | | | | | | |
| 4 | randomised trials | serious^i^ | serious^j^ | not serious | serious^d^ | none^b^ | 104 | | 93 | | - | SMD **0.3 lower** (1.22 lower to 0.61 higher) | ⨁◯◯◯ Very low | CRITICAL |

**CI:** confidence interval; **MD:** mean difference; **SMD:** standardised mean difference

**Explanations**

a. Some trials had problems with randomization process, deviations from the intended interventions, missing outcome data, measurement of the outcome, and selection of the reported result.

b. The publication bias was not detected due to the number of studies less than 10.

c. Some trials had problems with randomization process, deviations from the intended interventions, missing outcome data, and selection of the reported result.

d. Wide 95%CI with small sample size.

e. Some trials had problems with randomization process and selection of the reported result.

f. The I^2^ = 79%, P = 0.009.

g. Small sample size.

h. The I^2^ = 65%, P = 0.06.

i. Some trials had problems with randomization process, deviations from the intended interventions, and selection of the reported result.

j. The I^2^ = 89%, P < 0.001.

1. **Caregiver burden**


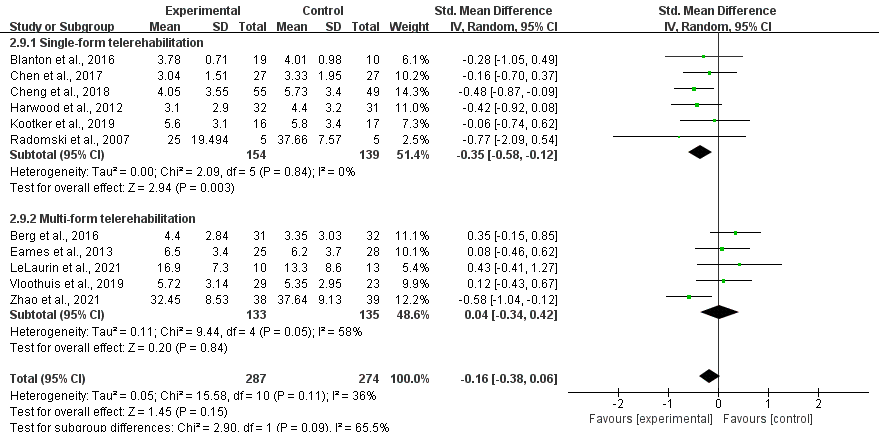


1. **Depression**


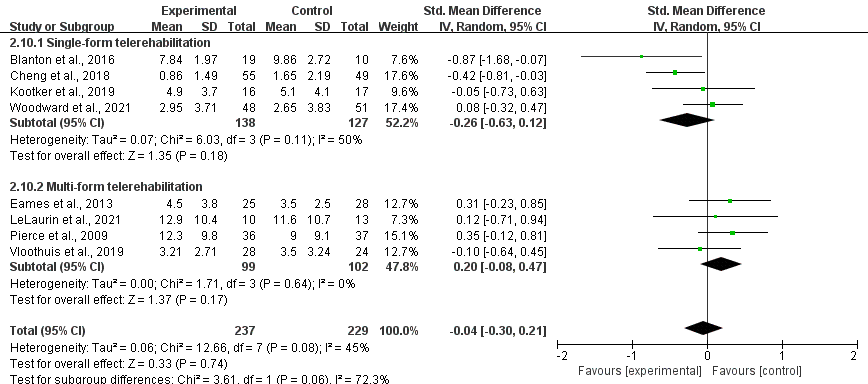


**Supplementary Fig.2. Subgroup analysis.**
